# Endocrine, genetic, and microbiome nexus of obesity and potential role of postbiotics: a narrative review

**DOI:** 10.1007/s40519-023-01593-w

**Published:** 2023-10-20

**Authors:** Weiming Wu, Zhengfang Chen, Jiani Han, Lingling Qian, Wanqiu Wang, Jiacai Lei, Huaguan Wang

**Affiliations:** 1 Department of Endocrinology, Changshu Hospital Affiliated to Nanjing University of Chinese Medicine, Changshu, 215500 Jiangsu People’s Republic of China; 2 Department of Endocrinology, Changshu First People’s Hospital, Changshu, 215501 Jiangsu People’s Republic of China; 3 Department of Gastroenterology, Hangzhou Ninth People’s Hospital, Hangzhou, 310005 Zhejiang People’s Republic of China

**Keywords:** Obesity, Energy metabolism, Gut microbiome, Postbiotics, SCFAs, Muramyl di-peptides, Bacteriocins, LPS

## Abstract

Obesity is a public health crisis, presenting a huge burden on health care and the economic system in both developed and developing countries. According to the WHO’s latest report on obesity, 39% of adults of age 18 and above are obese, with an increase of 18% compared to the last few decades. Metabolic energy imbalance due to contemporary lifestyle, changes in gut microbiota, hormonal imbalance, inherent genetics, and epigenetics is a major contributory factor to this crisis. Multiple studies have shown that probiotics and their metabolites (postbiotics) supplementation have an effect on obesity-related effects in vitro, in vivo, and in human clinical investigations. Postbiotics such as the SCFAs suppress obesity by regulating metabolic hormones such as GLP-1, and PPY thus reducing feed intake and suppressing appetite. Furthermore, muramyl di-peptides, bacteriocins, and LPS have been tested against obesity and yielded promising results in both human and mice studies. These insights provide an overview of targetable pharmacological sites and explore new opportunities for the safer use of postbiotics against obesity in the future.

## Introduction

Over the decades, researchers have faced a global challenge in understanding preventing and treating obesity and its accompanying metabolic consequences. Obesity prevalence and its related metabolic conditions have skyrocketed worldwide, particularly in developed countries [1, 2]. Given the link between obesity and both short- and long-term poor somatic, psychological, and socioeconomic circumstances, various studies support the WHO's assessment that obesity is one of the most serious threats to global public health today [3–5]. The development of obesity is linked to several variables. Along with the genetics, hormonal, and environmental factors, the utilization of high-calorie junk foods, a high consumption rate, less physically demanding occupations, a lack of physical activities, insufficient sleep, and repeated use of some medications contribute significantly to the onset of obesity [6]. Obesity is a complex and heritable illness caused by the interaction of genetic predisposition, epigenetics, metagenomics, and the environment. Numerous genes related to syndromic monogenic, non-syndromic monogenic, oligogenic, and polygenic obesity have been found in attempts to understand the genetic basis of obesity [7]. The genetics of leanness are also regarded as important since they reflect some of the etiologies of obesity. Various studies have witnessed different genes linked to monogenic obesity in humans. The mutations in leptin (an adipocyte-specific secreted protein associated with energy expenditure and appetite), leptin receptor, melanocortin 4 receptor (a G-protein-coupled receptor involved in energy homeostasis), and prohormone convertase 1 (involved in prohormone management), defects in pro-opiomelanocortin precursor (precursor of adrenocorticotrophin, melanocyte-stimulating hormone) [8]. The variations in these genes were also associated with severe consequences including a defective immune system, cardiovascular diseases, insulin resistance, metabolic dysfunctions, type-2 diabetes, ageing, and cancer [9]. The hormones secreted from the endocrine tissue, adipose tissue, and neuroendocrine cells mediate appetite, body composition, and glucose homeostasis [10, 11].

Improper nutrition not only affects the composition and function of the gut microbiota, but it also has a direct impact on energy intake and can contribute to the development of obesity [12]. The neural system regulates energy expenditure through the stimulants from the gastrointestinal tract in the form of neurotransmitters and other neuropeptides generated by gut microbiota [7, 13]. The regulatory chemicals generated by the microbiota have an impact on brain areas that are in charge of cognitive processes, emotions, and food consumption [14]. In obesity, the negative energy balance (due to increased physical activity or decreased food consumption, or both) is important concerning energy expenditure, physical and metabolic activities, and orexigenic signals [15].

The fact that probiotics and their metabolites play an important role in maintaining health and help in treating and mitigating various gastrointestinal (GIT) diseases/conditions via maintaining intestinal homeostasis cannot be ignored [16, 17]. “Postbiotics” is a term used to describe biological components produced by probiotics that have beneficial effects on the host. These biological components such as short-chain fatty acids (SCFAs), bacteriocins, lipoteichoic acids, surface layer protein, and secreted protein were named postbiotics in recent scientific discoveries. [18–21]. It is worth noticing that the host microbiota varies among individuals and populations and so as well as its metabolites, which are linked to the difference in functional phenotype as well as the metabolic status of the host [22]. The use of SCFAs and other microbial compounds produced by the host’s gut microbiota may also explain the intricacy of the pathogenic pathways linked to obesity [23, 24]. Therefore, this review aims to describe the physiology and molecular mechanism that directly and indirectly lead to obesity, furthermore, highlighting the nutritional strategy of using postbiotics and its action mechanism in encountering obesity and weight gain.

## Current global situation of obesity

The body mass index (BMI) scale is the most widely used to assess obesity [25]. According to the World Health Organization, BMI is “a basic indicator of weight-for-height that is routinely used to classify adults as underweight, overweight, or obese”. The most recent report published by WHO (2022) obesity is an emerging epidemic in developed and developing countries worldwide. The WHO fact sheet (https://worldpopulationreview.com/country-rankings/obesity-rates-by-country) about obesity updated in 2022 reported about 39% of the adult population aged 18 and above as obese, while those lower than 18 has a rise of 18% in 2016 compared to the 4% in 1975.

Obesity and its consequences are important factors contributing to morbidity, mortality, and compromise living standards, its complications can have a major effect on the financial and social life of an individual and population [26]. As it is strongly linked with mortality due to high-risk diseases such as cardiovascular, liver diseases, and certain types of cancer [26–28]. A recent report by “Statista, 2022” (https://lb-aps-frontend.statista.com/statistics/1287734/rate-of-deaths-attributable-to-obesity-leading-countries-worldwide/) showed that the mortality credited for obesity is 62.6 per 100,000 population.

## Obesity and energy metabolism linkage/consequences

In the recent era of industrialization, easy transportation, urbanization, and developments, a significant decline in physical activities leading to an imbalance of energy homeostasis cannot be ignored [29, 30]. These factors hugely favoured the condition of obesity and increased body weight by an easily and increased food access. Energy homeostasis refers to the intake of energy compared to its expenditure within the frontiers of thermodynamics law [31, 32]. A persistent positive energy results in obesity, it just takes a 1% increase in daily energy consumption for the average person to accumulate a 10-kg gain in fat mass over a decade [32, 33]. The energy intake and energy expenditure balance maintain the whole body’s energy homeostasis, when this balance is disturbed due to the contemporary lifestyle tied to the energy rich diet, the surplus energy is stored in the form of adipose tissue leading to obesity [34]. Obesity causes increased circulation of free fatty acids (FFA), which in turn induces oxidative stress by stimulating the reactive oxygen species (ROS) [35]. The elevation in ROS is the factual cause of insulin resistance. The decrease in liver antioxidant enzymes glutathione (GSH) is strongly linked with a high-fat diet (HFD), whereas the NADPH oxidase, which is involved in (ROS) production, is increased [36, 37]. Markers of oxidative stress increase in skeletal muscle because of HFD, which induces peripheral insulin resistance and ectopic fat storage [38, 39]. Over time, the pancreas gets exhausted and blood glucose levels begin to rise because there is not enough production of insulin to overcome the resistance. Once hyperglycemia occurs, the toxic effect on islet cells (glucotoxicity) intensifies the problem, thus lipotoxicity takes place as a result of increased FFAs levels [26, 40]. Insulin resistance in the liver, muscles, and adipose tissue escalates proinflammatory cytokines and de-escalates anti-inflammatory cytokines, which results in chronic inflammation [26]. No wonder how risky is obesity, and its consequences, as it is significantly associated with life-threatening diseases such as cardiovascular diseases, type two diabetes, cancer, osteoarthritis, and liver diseases [26, 27]. These risks arise from the enlarged number of adipocytes formation and their metabolism. Considering these consequences, obesity increases overall mortality, which needs serious attention.

## Endocrinal regulation of obesity

The hormonal imbalance and its resulting abnormalities are significantly associated with obesity [41]. The lean body maintains the normal regulation of the endocrinal system, an increase in weight causes the disproportion of several hormones and affects normal physiology [14, 41]. The hormones secreted from the endocrine tissue, adipose tissue, and neuroendocrine cells mediate appetite, body composition, and glucose homeostasis [10, 11]. These hormonal signals are strictly controlled in order to keep body weight/adiposity within a restricted, individually determined range, which can be influenced by factors such as calorie intake, meal composition, and lifestyle [10, 42]. In response to changed energy balance, the hypothalamus analyses and integrates a variety of neuronal and humoral cues to coordinate eating and energy expenditure. Long-term signals from the hypothalamus provide information about the body’s energy resources, an endocrine condition, and overall body condition [43]. Meal initiation and termination are supervised by short-term cues such as gut hormones and neurological impulses from the brain centre and gut. Both these short-term and long-term cues significantly influence energy expenditure by affecting sympathetic nerve outflow to brown adipose tissue and pituitary hormone release [44, 45]. Parallel to the neurological system’s control of appetite, the gut–brain axis communicates continuously from the stomach to the brain in both health and sickness. Not only does the gut microbiota connect with adjacent cells, but also produces and releases chemicals that can communicate with distant cells [46, 47]. In this regard, any changes to it may have a significant effect on appetite control. Gut microbiota and their metabolites (postbiotics) target the central nervous system (CNS) directly through vagal stimulation or indirectly through immune–neuroendocrine processes. Indeed, fat tissues are metabolic/endocrine organ that secretes adipokines, chemokines, and proinflammatory cytokines such as tumour necrosis factor-alpha (TNF-α), and interleukin-6 (IL-6), and others, thus play an important role related to obesity, and inflammation [48]. The adipose tissue releases three major hormones leptin, adiponectin, and visfatin [10]. Leptin is released by the white adipose tissue according to the body fat mass which induces an anorexigenic reaction and increases the expenditure of energy [49, 50]. The administration of leptin both peripheral and central significantly reduced the feed intake and feeding behaviour in mice [51]. Six alternative splice isoforms have been identified yet, and Ob-Rb among them is found high in the hypothalamus and other cells and acts as a primary signal transducer in the JAK–STAT signalling pathway [52]. Overall leptin acts as a mediator for energy homeostasis, through blood glucose regulation, feed intake, and eating behaviour in humans and mice [53, 54].

Adiponectin regulates insulin and acts as an anti-inflammatory agent, which is reduced in obese conditions [28]. The synthesis of adiponectin is triggered by glucocorticoids, prolactin, growth hormone, and catecholamine, while inhibited by androgens and the paracrine actions of TNFα [55, 56]. Adiponectin stimulates glucose metabolism and fatty acid oxidation in muscle tissue [57], while in the liver it increases insulin sensitivity, limits non-esterified fatty acids inflow stimulates fatty acids oxidation, and minimizes glucose synthesis and release [58]. A study performed in adiponectin knockout mice showed reduced hepatic insulin sensitivity and glucose intolerance [59].

Visfatin is an insulin-like peptide hormone generated by adipocytes that stimulates glucose absorption in muscles and skin while blocking its release from the liver [60]. A study in mice revealed visfatin lower glucose levels in an insulin-independent mode [60, 61]. Visfatin promotes the accumulation of triglycerides from pre-adipocytes, enhances glucose to lipid conversion, and upregulates the expression of various genes including PPAR gamma and adiponectin. However, visfatin did not alter the food intake or body weight in a knockout heterozygous mouse compared to the wild type [60–62]. Besides these, there are other hormones like insulin, ghrelin, obestatin, and so on (Table 1) which directly or indirectly affect body weight showing a deep connection between endocrinology and obesity.

**Table 1:** The tissue localization and characteristics of important hormones related to obesity and energy metabolism

Hormone	Localization	Function	Study	References
Leptin	Adipose tissue	Glucose, insulin regulation/increased energy expenditure	Human / mice	[63, 64]
Adiponectin	Adipose tissue	Insulin regulator/ anti-inflammatory	Human /mouse	[65, 66]
Visfatin	Adipose tissue	Glucose/insulin regulation	Human /mouse	[61, 67]
Insulin	Pancreatic islets	Fasting/feeding/lipogenesis	Human /mouse	[68, 69]
Ghrelin	Oxyntic glands/ gastric mucosa	Fasting/feeding/lipogenesis	Human /mouse	[29, 70]
Obestatin	Gastric mucosa	Suppress fasting	Human /mouse	[70, 71]
Cholecystokinin	Intestine/ hypothalamus	Suppress fasting, increase intestinal motility, stimulate the pancreas	Human /mouse	[72, 73]
Glucagon-like peptide-1	Intestine	Suppress appetite, increase energy expenditure, decrease intestinal motility	Human /mouse	[29, 74]
Polypeptide YY	Intestine/ileum	Suppress feed intake, glucose homeostasis	Human /mouse	[29, 75]
Glucose-dependent insulinotropic polypeptide	Upper intestine	Adipose regulation, glucose homeostasis	Human /mouse	[76, 77]
Oxyntomodulin	Intestine	Suppress appetite, feed, and intake, increase energy expenditure	Human /mouse	[78, 79]
Secreted frizzled related protein-5 (Sfrp-5)	Adipose tissue	Glucose/insulin/ lipid regulation	Human /mouse	[80, 81]

## Genetics of obesity

The genetic contribution to obesity cannot be underestimated due to the significant heritability of the BMI (20–40%) [82, 83]. Evidence showed that there has been a considerable link between genetics and obesity, with two studies claiming a heritability value of 0.77 at different ages in different regions [84, 85]. To date, there have been discoveries of some important genes strongly related to severe obesity, which give enough evidence of genetic and obesity linkage, as shown in Table 1. Recently, through GWAS combined with the in vivo study in *C. elegans*, scientists discovered 14 genes that promote obesity and 3 genes that prevent diet-induced obesity as shown in Table. 2 [86]. Referencing the studies performed previously several hundred genetic loci have been found by genome-wide association analysis (GWAS) studies, where sequence variants are statistically linked with BMI at the population level, however, these links show only a 3–5% contribution of variation to the BMI [82, 87, 88]. Furthermore, the majority of obesity-predisposing gene variations are not linked to weight loss or regain due to lifestyle interactions.

**Table 2:** The genomic information of the obesity-related genes, and their functional characteristics

Gene	Full name	Chr. location	Consequences	Study	Associated traits	References
FTO	Fat mass-and obesity associated gene	16q12.2	Severe obesity	Human/mice	Promote food intake	[96, 97]
LEP	Leptin	7q32.1	Severe obesity	Human/mice	Hyperphagia, metabolic, immune dysfunction, hypogonadism	[63, 98]
LEPR	Leptin receptor	1.p31.3	Severe obesity	Human/mice	Hyperphagia, metabolic, immune dysfunction, hypogonadism	[63, 98]
MC4R	Melanocortin 4 receptor	18q21.32	Severe obesity		Involve in growth development and growth hormone, hyperinsulinemia	[63, 99]
PCSK1	Proprotein convertase subtilisin/kexin type 1	5q15	Child obesity	Human	Involve in glucose homeostasis, hyperphagia, decreased growth, hypothyroidism, hypocortisolism, and hypogonadotropic hypogonadism	[100, 101]
BDNF	Brain-derived neurotrophic factor	11p14.1	Severe early obesity	Human/mice	Severe obesity, hyperphagia, impaired cognitive	[102, 103]
KSR_2_	Kinase suppressor of ras 2	12q24.22-q24.23	Child obesity	Human/mice	Hyperphagia, insulin resistance, reduced metabolic rate	[104, 105]
POMC	Proopiomelanocortin	2p23.3	Child obesity	Human	ACTH, red hair, and pale skin	[106, 107]
ADCY3	Adenylate cyclase 3	2p23.3	Early obesity	Human/ mice	T2D	[108, 109]
ADIPOQ	Adipocyte-C1q, and collagen domain containing	3q27.3	Obesity	Human	Promotes energy and expenditure	[9, 110]
INSIG2	Insulin-induced gene 2	2q14.1-q14.2	Obesity	Human	Involve in cholesterol regulation and fatty acid synthesis	[111, 112]
PPARG	Peroxisome proliferator-activated receptor gamma	3p25.2	Obesity	Human	Stimulate and development of fat tissue	[113, 114]
ADCY3	Adenylate cyclase	12A1.1	Obesity	Human/ mice	Obesity, diabetes, and energy metabolism	[96, 115]

### Genetics of obesity studies conducted in humans and mice model

In order to improve the prevention, treatment, and management of obesity it is important to undermine and understand its molecular causes. Consequently, this has encouraged identifying the genes responsible for obesity using rodent and human models [40, 89]. The mouse model is widely used in studying the genetics of obesity due to its low cost, maintenance, small size, easy breeding, and short gestation period [90–92]. The complete genome sequence, genetically distinct strains availability, and cutting-edge genetic manipulative tools make it possible to conduct advanced genetic analysis associated with obesity in rodents. Furthermore, the occurrence of obesity and metabolic phenotypes alteration in mice are similar to humans, moreover, the measurement of these phenotypes in mice is more convenient and safe compared to humans [93, 94]. However, there are certain limitations in the mouse model used for obesity phenotypes compared to the human model, such as the difference in obesity phenotypes, and physiology, which leads to further and safe investigation in the human model [95]. From the literature, we have identified several genes that are directly related to obesity and both verified in mice and human models as shown in Table 2.

### Novel genes in human obesity using the *C. elegans* model

Previously genetic selection using the *Caenorhabditis elegans* model has led to the discovery of drug targets for various diseases including depression and metabolic related disorders [116, 117]. *C. elegans* is considered, evolutionarily distant from humans, as the many pathways related to lipid, glucose, and protein metabolism are the same in both species [118]. In both organisms, identical genes such as TOR kinase and AMPK, as well as transcription factors like sterol response element binding protein (SREBP), peroxisomes proliferator-activated receptor gamma (PPAR), and transcription factor EB (TFEB), govern metabolic genes and cellular responses. Studies have also shown that the loss of function of such regulators in both species causes metabolic dysfunction [119–122]. Furthermore, obesity-related genes identified in human GWAS whose orthologue has been shown to contribute to obesity in *C. elegans* are more likely to be a robust anti-obesity target across human populations. Recently scientists discovered 11 novel and overall 16 genes as shown in Table 2, which promote or prevent *C. elegans* obesity, as well as the early beginning of organismal degradation and mortality linked with obesity [86]. The findings of in vivo research in *C. elegans* combined with assessments of mouse and human GWAS datasets revealed that the sign of the connection between the mouse and human gene expression levels and their associated clinical characteristics matched. The behavioural consequences of knocking down these genes in *C. elegans* revealed that these obesity genes had conserved causation and therapeutic potential [86] (Table 3).

**Table 3:** The molecular characteristics of the novel genes discovered in *C. elegans* involved in human obesity

Gene in *C. elegans*	Chr. location	Exon count	Human orthologue	Knock-out results
puf-8	Chr. II	6	Pumilio RNA binding family 2 (PUM2)	Promotes obesity
rpac-19	Chr. III	3	RNA polymerase I and III subunit D (POLR1D)	Promotes obesity
Fbf-2	Chr. II	8	Pumilio RNA binding family 2 (PUM2)	Promotes obesity
Gon-1	Chr. IV	31	ADAM metallopeptidase with thrombospondin type 1 motif 20	Promotes obesity
Glp-1	Chr. III	9	Notch receptor1 (NOTCH1	Promotes obesity
Hlh-2	Chr. I	6	Transcription factor (TCF-2)	Promotes obesity
Let-767	Chr. III	5	Hsd17b12	Prevent obesity
Pop-1	Chr. I	4	lymphoid enhancer-binding factor 1 (LEF1)	Promotes obesity
Mys-1	Chr. V	5	Lysine acetyltransferase 5 ( KAT5)	Promotes obesity
Zfh-2	Chr. 102C3-102C4; 4–0 cM	15	Zinc finger homeobox 3 (ZFHX3)	Promotes obesity
Nst-1	Chr. II	5	G-protein nucleolar 3 like (GNL3L)	Promotes obesity
Y46e12bL.2	Chr. II	9	Ribosomal RNA processing 12 homologs (RRP12)	Promotes obesity
Rpt-5	Chr. I	4	Proteasome 26S subunit, ATPase 3 (PSMC3)	Promotes obesity
Y71H10B.1	Chr. X	11	5′-Nucleotidase, cytosolic II (NT5C2)	Prevent obesity
pho-1	Chr. II	11	ACP-2	Prevent obesity
Eif-6	Chr. I	3	Eukaryotic translation initiation factor 6 ( EIF6)	Promotes obesity

### Epigenetics of obesity

**Fig. 1:**
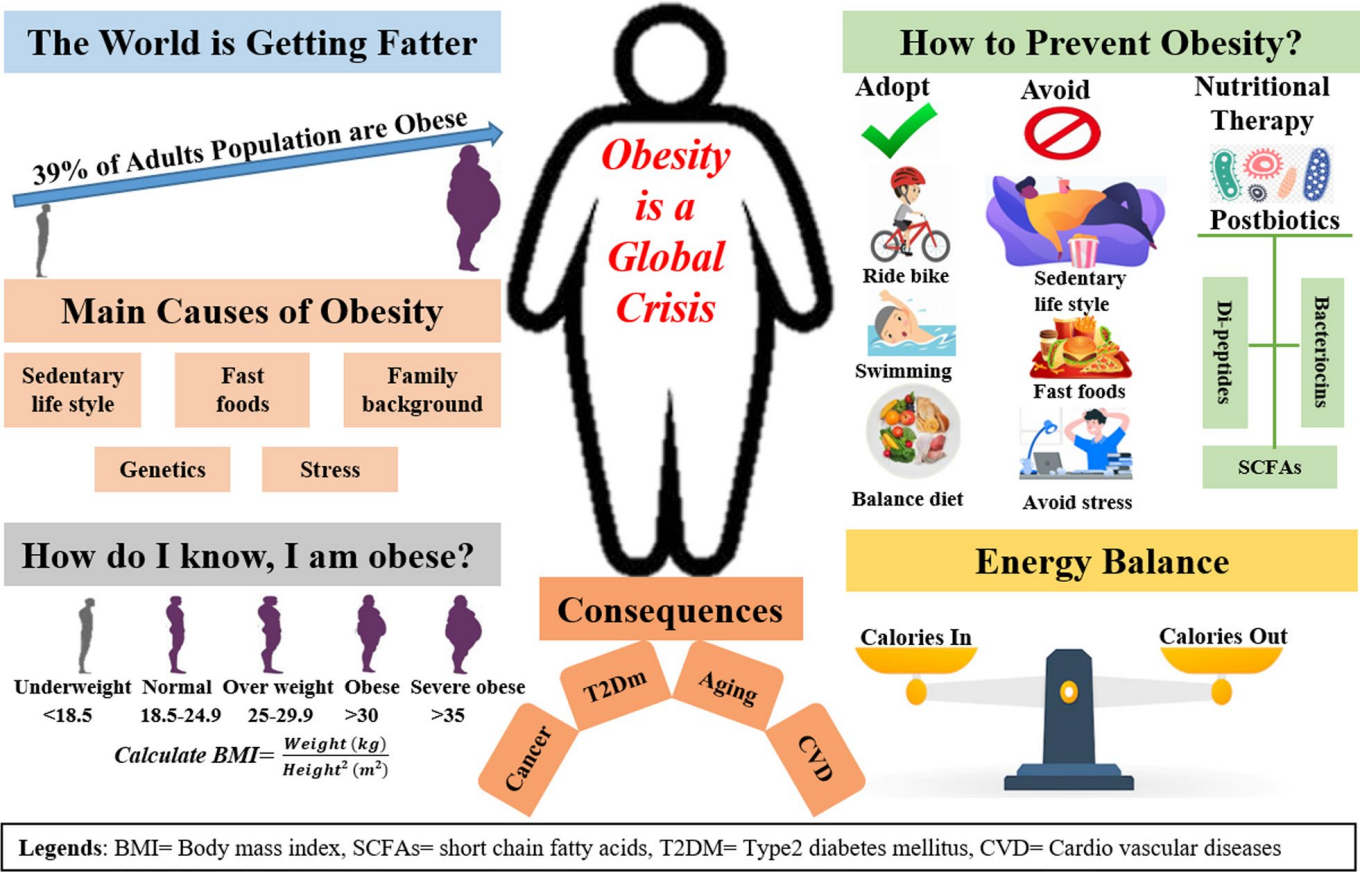
Environment, genetics, and epigenetics contribute as the main factors causing obesity. Environmental factors such as a sedentary lifestyle, unhealthy food, stress, and abnormal sleep along with genetics and epigenetics are predisposing causes for obesity. Both these factors cause epigenetics alteration, which causes energy dysbiosis, tissue inflammation, decrease insulin resistance, and increase lipid accumulation. In turn, obesity is capable of causing severe health problems such as cancer, type-2 diabetes, ageing, and cardiovascular disease are the most common

For the development of novel obesity causing DNA variations, the duration of the obesity as a pandemic is not long enough. In this case, the dynamic epigenetic regulations and environmental factors are leading contenders for explaining energy regulations [6, 7]. So far, DNA methylation has been the most thoroughly researched epigenetic mark for human disorders at the genome-wide or site-specific level, which takes place at the cytosine [83, 88, 123]. Various studies have identified the methylated loci through the epigenetic wide association studies linked with obesity. Furthermore, these studies uncovered that several genes had undergone methylation associated with obesity [124]. CpG promoter methylation of peroxisome proliferator-activated receptor gamma (PPARc) coactivator-1alpha (PGC-1a), a transcriptional coactivator for mitochondrial biogenesis, is elevated in obese women's subcutaneous adipose tissue (SAT) [88]. In obese people, adiponectin, an adipokine that controls systemic energy expenditure and insulin sensitivity, is diminished in adipose tissue [125]. In very obese patients, DNA methylation levels at the adiponectin gene locus in SAT were linked to BMI. The hypothalamus through regulation of the pro-opiomelanocortin (POMC) gene controls energy homeostasis, and methylation in the POMC gene was found to be significantly associated with obesity. Only a few particular genes and pathways have been consistently identified as being involved in the development of obesity [6]. Therefore, despite the genetic susceptibility to obesity, environmental and epigenetics seem to be important factors (Fig. 1).

## Gut microbiota and obesity linkage

The gut microbiota plays an important role in the development of obesity due to its intimate nexus with energy metabolism. Any alteration in gut microbiota may lead to energy dysbiosis as well as energy homeostasis [14, 126]. The human gut hosts a diverse microbial population among which ~ 1000 bacteria are preponderant and belong to 40 different species [16, 127]. In order to disclose the predictive microbial markers of obesity, the Firmicutes phyla’s staphylococcus and lactobacillus, as well as Bifidobacterium from the Actinobacteria genus, were examined. Interestingly Bifidobacterium showed a higher number in normal-weight compared to the obese individuals, while staphylococcus were less [128, 129]. With the decrease of keystone microbial species in the guts, the symbiosis between the host and gut microbiota is disturbed, resulting in dysbiosis, which unsettles the host's metabolic health. On one side dysbiosis is considered to be a result of a decreased number of bacteria that are metabolically protective against obesity, and an increase in those that extract more energy from indigestible carbohydrates. Furthermore, various studies including clinical trials suggested that intervention of certain microbial species exerts a significant effect and mitigates obesity as shown in Table 4.

**Table 4:** Clinical trials signifying important microbial species alone or in combination involved in obesity mitigation

Microbial specie	Dose CFU/day	Trial duration	Results	References
L. gasseri BNR17	10^10^	12 weeks	↓ Body fat	[130]
L. gasseri SBT2055	10^6–8^	12 weeks	↓Body weight	[131]
L. salivarius UCC18	10^9^	4 weeks	↓ Body weight	[132]
L. paracasei F19	9.4 × 10^10^	6 weeks	Improved insulin sensitivity	[133]
B. breve B-3	5 × 10^10^	12 weeks	↓ Body fat	[134]
B. animalis, lactis B420	10^10^	6 months	↓ Body fat	[135]
L. rhamnosus CGMCC1.3724	3.24 × 10^8^	12 weeks	Appetite control	[136]
L. plantarum KY1032, and L. curvatus HY7601	2.5 × 10^9^	12 weeks	↓ Body weight	[137]
B. breve B-3	2 × 10^10^	12 weeks	↓ Body fat	[134]
B. lactis CECT 8145	10^10^	12 weeks	↓ Weight	[138]
B. pseudocatenulatum CECT 7765	10^9–10^	13 weeks	↓ Weight	[138]

Various studies using the animal model have proposed that the gut microbiota energy homeostasis and adiposity through various mechanisms. The gut microbiota extracts energy from the diet while also modifying tissue fatty acid composition, secreting gut-derived peptides and hormones with CNS effects, and generating chronic low-grade inflammation via lipopolysaccharide release [48, 129]. One of the critical tasks of the gut microbiota is the enzymatic conversion of primary bile into secondary bile, which influences the absorption and emulsification of bile acids. Following this mechanism gut microbiota has an enormous impact on bile acid entero-hepatic distribution. The secondary form binds to G-protein and leads to glucagon-like-1 peptide stimulation, which lowers circulation and hepatic triglyceride levels [126, 139]. Both qualitative and quantitative variations in the gut microbiota can affect this pathway by encouraging fat formation in the body. For instance, gut microbiota on a high-fat diet may convert dietary choline into hepatic toxic methyl-amines, lowering choline availability, which is required for very low-density lipoprotein (VLDL) assembly and production, thus significantly enhancing hepatic steatosis and lipo-peroxidation. The intestinal microbial community plays an important role in the processing of dietary carbohydrates, and their fermentation into SCFAs. The acetic acid that is the most abundant in peripheral blood is vital for cholesterol synthesis, a stimulant for adipogenesis via the FFA2 receptor, and a suppressor of appetite via the hypothalamic mechanism. Propionic acid is the main precursor for protein synthesis, hepatic gluconeogenesis, and lipogenesis, as well as an inhibitor of fatty acid production, and an inflammation-reducing agent [16, 48, 126].

## Host and gut microbial metabolites (postbiotics) interaction

The intestinal microbial community plays an important role in the processing of dietary carbohydrates, and their fermentation into microbial metabolites. Growing findings suggest that microbial metabolites (postbiotics) produced by microbial fermentation have an important role in regulating host metabolism, with implications for obesity [13, 135]. Clostridium and Eubacterium from the gut microbiota convert bile acid in the intestine to secondary forms such as deoxycholic acid and lithocholic acid, which bind to the TGR5 receptor (G-protein-coupled receptor) and stimulate the secretion of the incretin hormones GLP-1 and insulin, promoting energy expenditure [140]. Long chain fatty acids produced by gut microbiota, such as linoleic acid, modify the lipid profile, contributing to obesity. The acetic acid that is the most abundant in peripheral blood is vital for cholesterol synthesis, a stimulant for adipogenesis via the FFA2 receptor, and a suppressor of appetite via the hypothalamic mechanism. Propionic acid is the main precursor for protein synthesis, hepatic gluconeogenesis, and lipogenesis, as well as an inhibitor of fatty acid production, and an inflammation-reducing agent [16, 48, 126].

### Short-chain fatty acids in control of energy regulation

Due to a lack of suitable enzymes, our gut bacteria ferment dietary components that are incompletely hydrolyzed, leading to the formation of SCFA such as acetate, butyrate, and propionate [141]. These SCFAs have important roles in the pathophysiology of obesity and related illnesses by regulating energy intake, energy harvesting, and host energy and substrate metabolism, all of which affect body weight [142–145]. Several pathways have also been hypothesized to link SCFA to insulin sensitivity and the progression of T2DM, including interorgan effects on adipose tissue function and lipid storage capacity, metabolism, and inflammatory activities [146–148]. SCFAs are monocarboxylic acids including acetate, lactate, propionate, and butyrate as the most abundant and common metabolites secreted by the gut microbiota [127]. These SCFAs are the main constituent of fibre fermentation because of gut microbiota and exert significant effects on host physiology, gut health, mucous production, promoting gut integrity, and protection of the gut epithelial [23, 149].

Evidence suggests that glucose is not the only source of energy utilized by the body. In addition, the body uses SCFAs and amino acids to carry out various physiological activities [141]. A study reported the involvement of butyrate and propionate in stimulating different gut hormones and reducing feed intake [150]. Propionate blocks lipogenesis by downregulating fatty acid synthase in the liver, while acetate is a lipogenic substrate, thus, the acetate/propionate ratio is thought to be critical for de novo lipogenesis. In addition, propionate and butyrate induce intestinal lipogenesis by upregulating the lipogenesis-related genes, thus mitigating obesity.

#### Acetate and obesity nexus

**Fig. 2:**
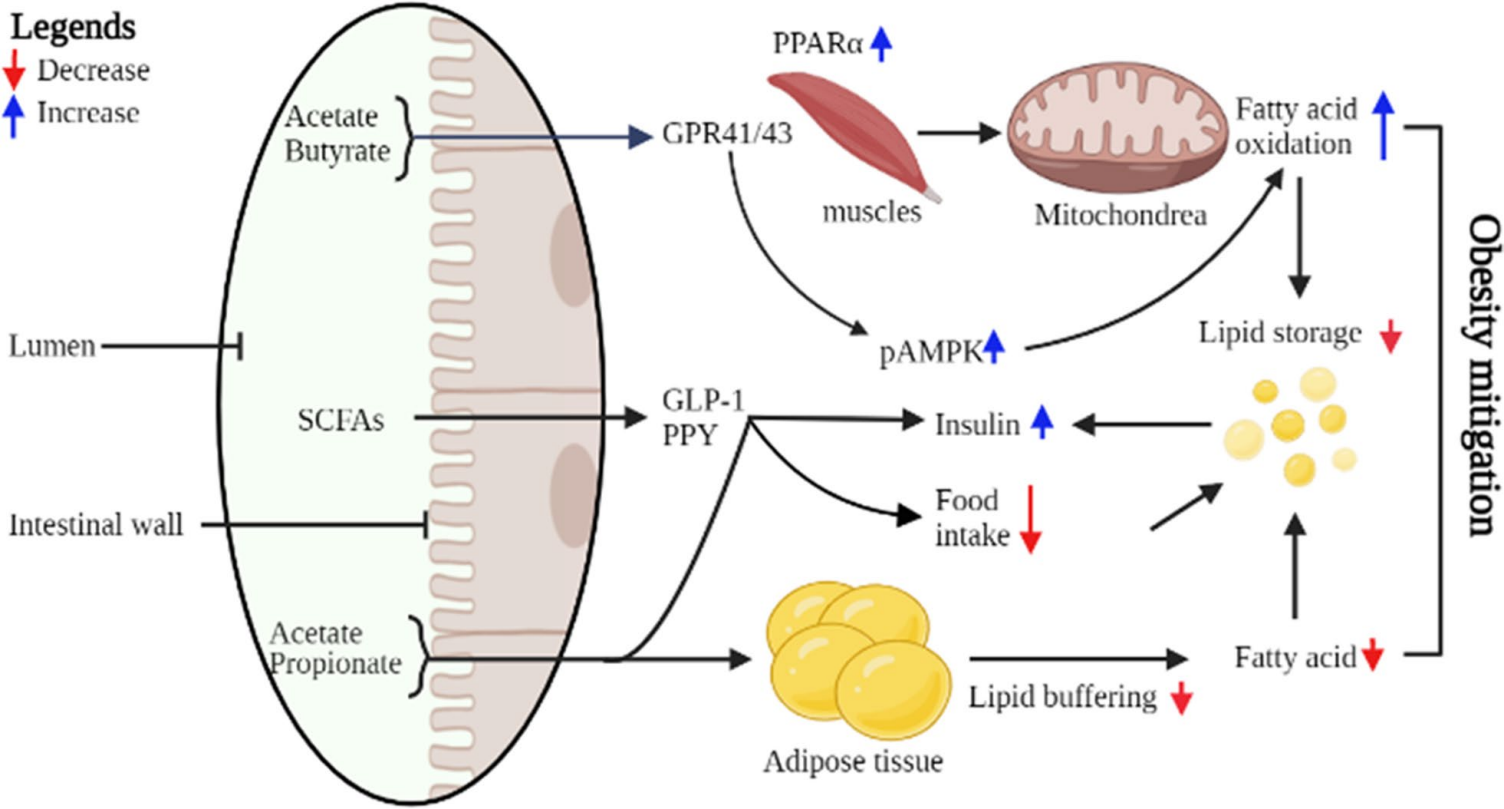
The molecular mechanism of SCFAs mitigating obesity through energy regulation. The intestinal wall absorbs acetate and butyrate in the intestine produced by the gut microbiota; regulate fatty acids, and insulin level through PPARα, GLP-1, and PPY signalling pathways

Acetate has been attributed to health benefits, whether derived from food or microbial fermentation in the gut. These health benefits include energy homeostasis, improved heart function, blood generation, and memory formation [151, 152]. The question of how acetate contributes to so many diverse biological functions is an area of intense research nowadays. Acetate is believed to be responsible for appetite regulation [153]. Supplementation of acetate can stimulate biochemical and physiological responses resulting in control of insulin regulation, weight loss, cardiac system safety, and anti-inflammatory responses [16]. Its function related to obesity is however still conflicting. On the one hand, acetate has been demonstrated to increase the expression of anorectic hormones in the hypothalamus, such as GLP-1 and peptide tyrosine-tyrosine (PYY), so decreasing food intake (Fig. 2) [14, 154] (Fig. 2). A study performed in mice showed that acetate generated in the intestine increases anorectic signalling in the arcuate nucleus ARC via the glutamate–glutamine transcellular cycle [154]. However, this statement was contradicted by another study that showed the increased level of acetate was involved in increasing insulin and ghrelin leading to obesity [155]. Therefore, further research is needed to confirm whether the acetate has a stimulating or suppressing effect on the appetite.

#### Propionate and obesity nexus

Propionate another SCFA has been reported to mitigate obesity and reduce feed intake through gut hormone modulation [153, 156]. Propionate suppresses the appetite by regulating free fatty acids receptor FFAR2/3 in the intestinal cells, which induces the glucagon-like peptide (GLP-1) and PPY peptides [157]. The presence of propionate in the hindgut activates the PPY and GLP-1 involved in reducing both feed intake and weight gain in obese individuals (Fig. 2) [153]. Furthermore, propionate has been reported in the suppression of the genes responsible for lipid synthesis. Various studies performed using the mouse model explained the mechanism of propionic acid in preventing obesity by inhibiting food intake, increasing insulin sensitivity, and energy expenditure [145, 153, 158].

#### Butyrate and obesity nexus

Among others, butyrate is one of the most used SCFAs used by the intestinal mucosa, as a primary source of energy [14, 142]. Dietary butyrate has been reported in insulin resistance and prevents diet-induced obesity in mice [142]. Furthermore, butyrate has also been involved in controlling weight by boosting energy expenditure through direct contact with skeletal muscle and inducing lipolysis in adipose tissue. Butyrate supplementation in the diet showed a significant reduction in diet-induced obesity and insulin resistance in obese mice models [143, 159]. SCFAs are the byproducts of bioconversion in the colon and play a major role in appetite regulation by boosting the release of anorectic gut hormones such as PYY and GLP-1 [153]. As a result, raising SCFA levels represents a viable target that could lower adiposity and weight in obese persons. A study in mice revealed that oral administration of sodium butyrate induces fat oxidation and energy expenditure leading to weight loss [23, 142]. Moreover, the microbiota transplant from human to mouse resulted in increased adiposity, decreased faecal SCFAs, and increased monosaccharide and disaccharide concentration after feeding a plant carbohydrate-rich diet compared to the one received microbiota from the lean individual [131]. These studies suggest that the microbes from the obese individual have lower capabilities to properly ferment and digest the polysaccharides compared to the microbiota from the lean individual. However, the molecular effect of butyrate needs further investigation due to its controversial position as it also acts as a substrate for energy in the host system. To explore the real scenario behind this controversy, the actual role of butyrate in the energy cycle shall be tested in different animals, while using equicaloric food in both control and test groups.

### Peptidoglycans as postbiotics linkage with obesity

Adipose tissue inflammation and insulin resistance are some of the main consequences of obesity, however, certain microbial components can significantly protect against these damages [144]. For example, the postbiotics from the proximal gut microbiota showed a significant role in preventing insulin resistance because of a high-fat diet [160, 161]. Peptides are the important component of the bacterial cell wall present in the form of peptidoglycan. A recent study using a mouse model showed that peptide-based postbiotics (muramyl-dipeptide) reduced insulin resistance and adipose tissue inflammation in obese conditions through nucleotide oligomerization 2 protein receptors [20]. NOD2 acts as a bacterial peptidoglycan sensor and its activation stimulates metabolic, inflammatory, and antimicrobial activities. Furthermore, NOD2 keeps the gut microbiota healthy [162]. A study in mice reported that NOD2 knockout mice developed obesity due to a high-fat diet and caused metabolic disturbances including hyperglycemia, hyperlipidemia, and glucose intolerance. These repercussions consequentially lead to the accumulation of adipocytes and lipid droplet formation in the liver [163]. A single dose of MDP-based postbiotics reduced glucose intolerance via interaction with NOD2 receptors without damaging the gut microbiome [20]. Further insight into the NOD protein and postbiotics interaction related pathways will explore the molecular mechanism of action of postbiotics and recognition of specific pharmacological sites for treating obesity.

### Bacteriocins’ role in obesity

Bacteriocins are ribosomal-synthesized heat-stable antimicrobial peptides produced by the gut microbiota, which show distinct characteristics related to their size, structure, and mechanism of action [22, 150]. It is well known that bacteriocins show broad spectrum and narrow spectrum antimicrobial activities, however, certain studies also showed that some of these species that produce bacteriocins play an important role in obesity and related metabolic activities [19, 150]. Recent studies have underlined the population of various microbiota that may be involved in obesity. A study claimed that the gut microbiota of genetically obese mice have a higher population of phyla Firmicutes and lower phyla Bacteroidetes [17, 24]. Other studies have established the role of a particular species or strain in obesity and T2D [17]. In germ-free mice, it was revealed that Enterobacter cloacae B29 produces endotoxins that cause obesity and insulin resistance [164]. Furthermore, Clostridium ramosum, previously shown to be enriched in patients with T2D, induced obesity in mice consuming a high-fat diet [165]. Gut bacteria that produce antibiotics with specific activity against some of these organisms may be beneficial for balancing metabolic health.

### Lipopolysaccharides and obesity

Lipopolysaccharides (LPS), a component of Gram-negative bacteria’s cell membrane, the function of which has been ambiguous, act as a triggering factor, causing low-grade chronic inflammation and the development of insulin resistance (IR) [19]. LPS produced in the gastrointestinal tract enters the blood by direct diffusion via increasing intestinal permeability or absorption and chylomicron inclusion. High fat consumption reduces the expression of the tight junction proteins zonulin and occludin, increasing the intestinal permeability of LPS, the causative cause of endotoxemia [19, 69]. LPS interacts with toll-like receptor 4 (TLR-4) in immune cells as well as target organs such as the liver and adipose tissue. Migration of active NF-κB to the nucleus stimulates the production of proinflammatory proteins as well as signalling pathways such as JNK, p38 MAPK, and ERK, which leads to insulin resistance and obesity [166]. Bifidobacterium infantis administration decreased colonic permeability and inflammation in mice, indicating that gut microbial makeup, in addition to food, influences intestinal permeability. High levels of saturated lipid consumption not only increase systemic exposure to potentially proinflammatory-free fatty acids and their derivatives but also enhance the absorption of endotoxins, resulting in greater plasma LPS levels known as “endotoxemia” [8]. Endogenous lipid interaction with cannabinoid receptors (CB1 and CB2) activates adenylate cyclase and also promotes secondary messengers implicated in the MAPK, ERK, and NF-κB pathways, causing inflammation and insulin resistance and eventually contributing to obesity. Additionally, circumstantial evidence suggests that LPS compromises the liver’s critical role in preserving the body’s glucose metabolism. It has been demonstrated that LPS-stimulated macrophages from the gingival sulci release TNF-α, IL -1, and IL-6 in animal models of periodontitis [167]. These cytokines and/or LPS from the gingival sulci may be transported throughout the body and engage TLR-4 receptors on Kupffer cells in the liver to release proinflammatory cytokines, which may lead to insulin resistance and glucose intolerance [168]. Another study concluded that LPS concentrations are an adequate molecular trigger for high fat diet-induced obesity and diabetes. Finally, via regulating insulin sensitivity, the LPS receptor, cluster of differentiation antigen 14 (CD14) determines the cutoff point at which metabolic disorders manifest [169]. These evidence suggest that LPS might contribute to host obesity by modifying intestinal permeability, resulting in endotoxemia, increased calorie supply, and endocannabinoid system (eCB) activation, as well as by modulating lipid metabolism by increasing lipoprotein lipase activity and lipogenesis [8, 170]. However, the molecular details remain to be elucidated, as there is a complex interaction between LPS-induced inflammation and obesity, which needs further research.

## Conclusion

The current knowledge and shreds of evidence explaining the current global situation, molecular mechanism inducing obesity, its prominent causes, and the potent role of postbiotics in mitigating obesity. Irregularities in energy homeostasis due to changes in gut microbiota, environment, genetics, and epigenetics are highly linked to obesity. Postbiotics like SCFAs, lipids, and bacteriocins interact with genetics, hormones, nutrition, and certain environmental conditions as potential anti-obesity agents. SCFAs like acetate, propionate, and butyrate have strong capabilities to counteract obesity by regulating metabolic hormones such as GLP-1, and PPY thus reducing feed intake and suppressing appetite. Given the severity and burden of the condition on the healthcare system, the need to identify pharmacological targets for the treatment of obesity and adaptation of smart nutritional strategies are needed to explore which might further overcome this scenario. Further research is needed to explore the exact molecular mechanism of action of postbiotics in mitigating obesity, which might answer most of the questions raised related to this scenario. With the increase in physical activities, the regulated range of energy balance can be achieved, and the internal molecular mechanism to maintain this energy homeostasis can be improved. Moreover, the adoption of a smarter diet will decrease the need for drastic dietary restrictions to avoid abrupt disturbances in the energy system.

## Data Availability

Not applicable.
